# Supplementary material for: Multiple modes of convergent adaptation in the spread of glyphosate-resistant Amaranthus tuberculatus
Source: Proc Natl Acad Sci U S A. 2019 Sep 30;116(42):21076–84. doi: 10.1073/pnas.1900870116 (PMC6800383; doi:10.1073/pnas.1900870116)
Supplement: Supplementary File [file pnas.1900870116.sapp.pdf]

PNAS
www.pnas.org

Supplementary Information for

Multiple modes of convergent adaptation in the spread of glyphosate-resistant Amaranthus tuberculatus

Julia M. Kreiner, Darci Ann Giacomini, Felix Bemm, Bridgit Waithaka, Julian Regalado, Christa Lanz, Julia Hildebrandt, Peter H. Sikkema, Patrick J. Tranel, Detlef Weigel, John R. Stinchcombe & Stephen I. Wright

Detlef Weigel
Email: detlef.weigel@tuebingen.mpg.de

This PDF file includes:

Figures S1 to S5
Tables S1 to S2

www.pnas.org/cgi/doi/10.1073/pnas.1900870116

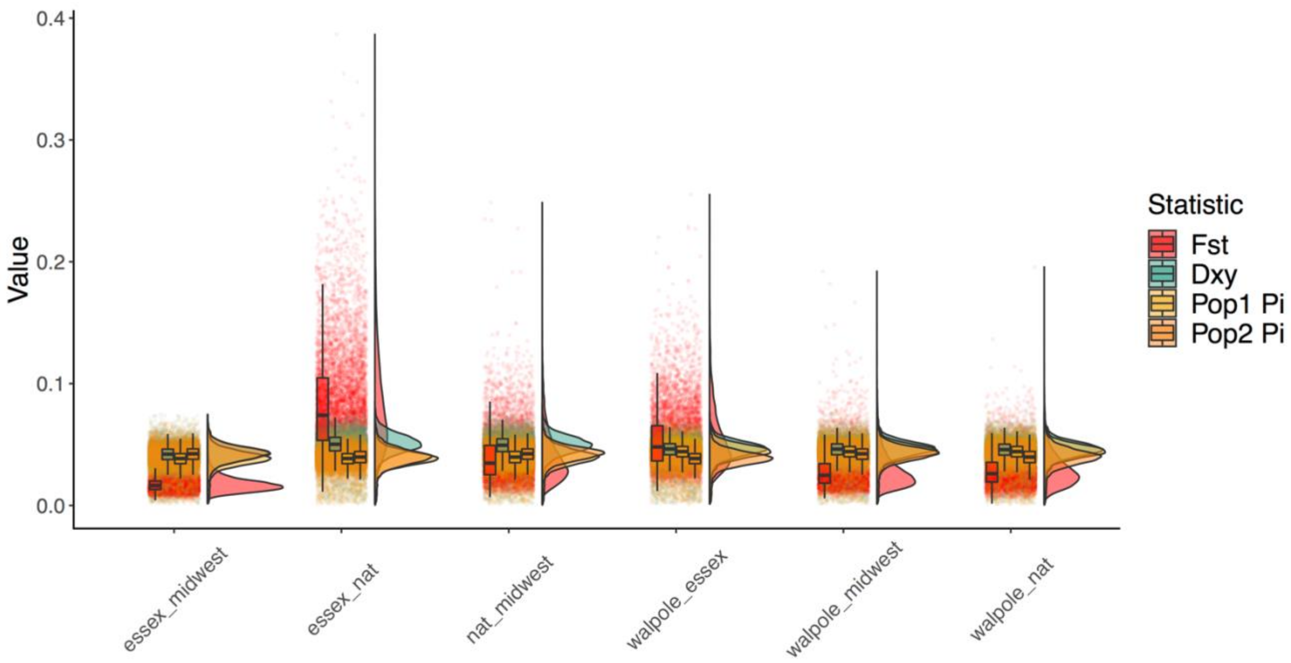

Fig. S1. The relationship between absolute diversity (D_{xy}), relative diversity (F_{ST}), and within-population diversities (π) for among geographic region comparisons. For each comparison, points refer to the means of 100 kb windows, with corresponding boxplots and density curves for each summary statistic. Pop1 in the legend refers to the first population of the pairwise comparison, and Pop2 to the second.

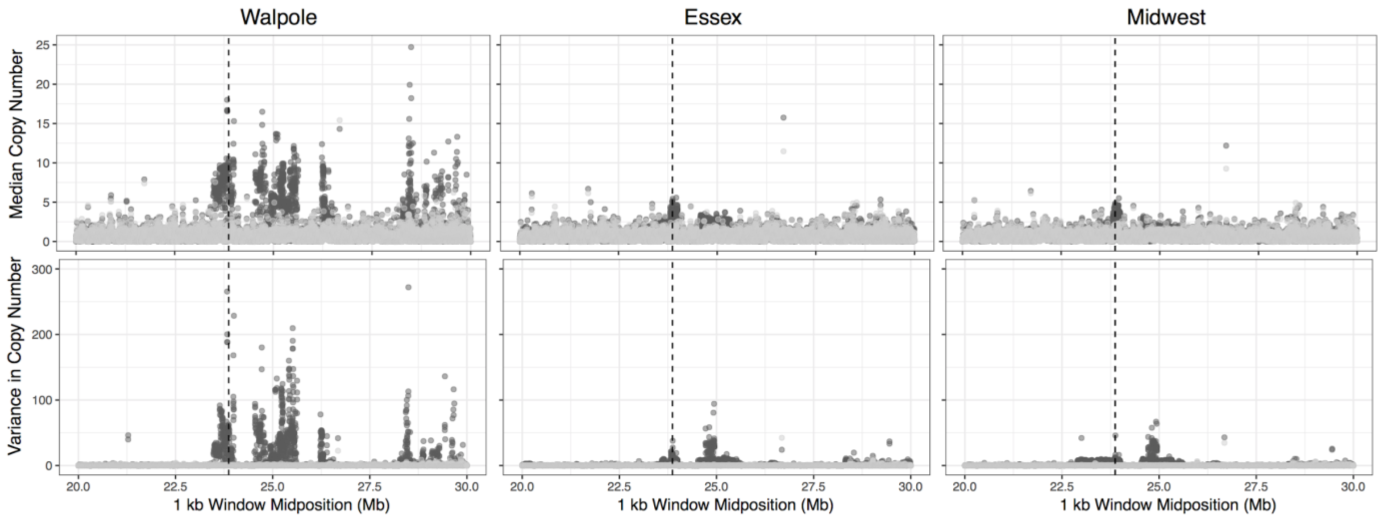

Fig. S2. Median and variance in *EPSPS* copy number for individuals with (dark grey) and without (light grey) copy number increase in chromosome 5. Dashed vertical lines indicate location of *EPSPS*.

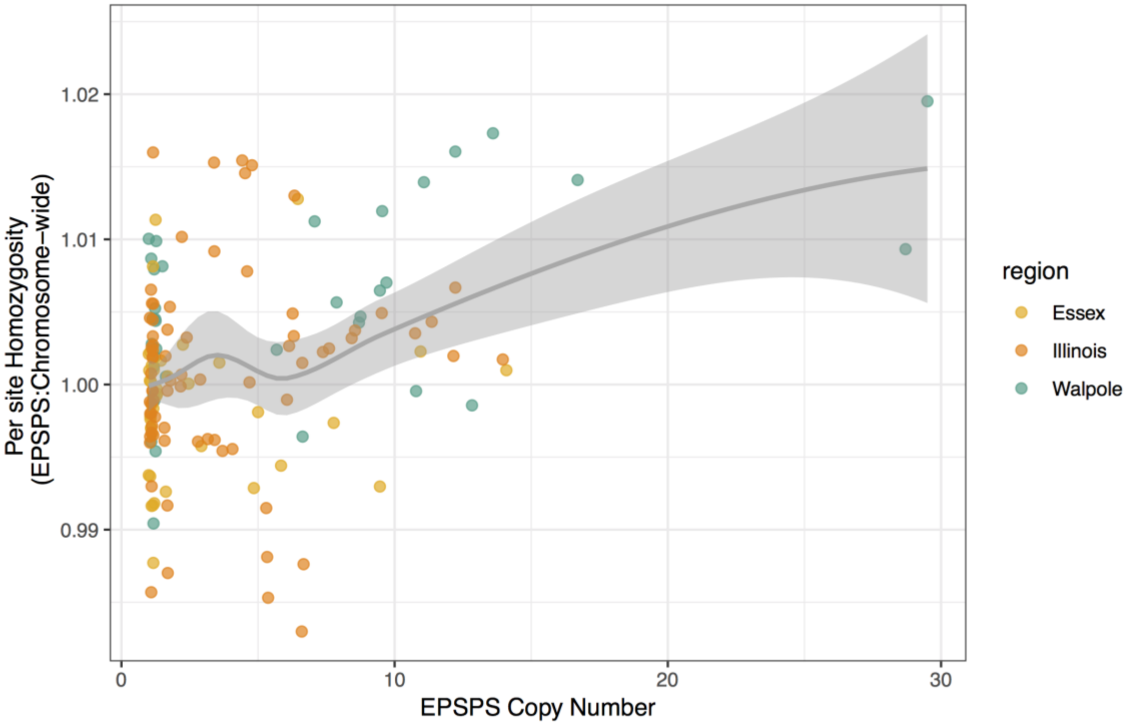

Fig. S3. The relationship between the ratio of *EPSPS* copy number and per-site homozygosity in 1 Mb around EPSPS relative to all of chromosome 5. An ANOVA for ratio of homozygosity to *EPSPS* copy number was significant, with increased copy number individuals showing significantly increased homozygosity (p = 5.2e-07 and r_2 = 0.147).

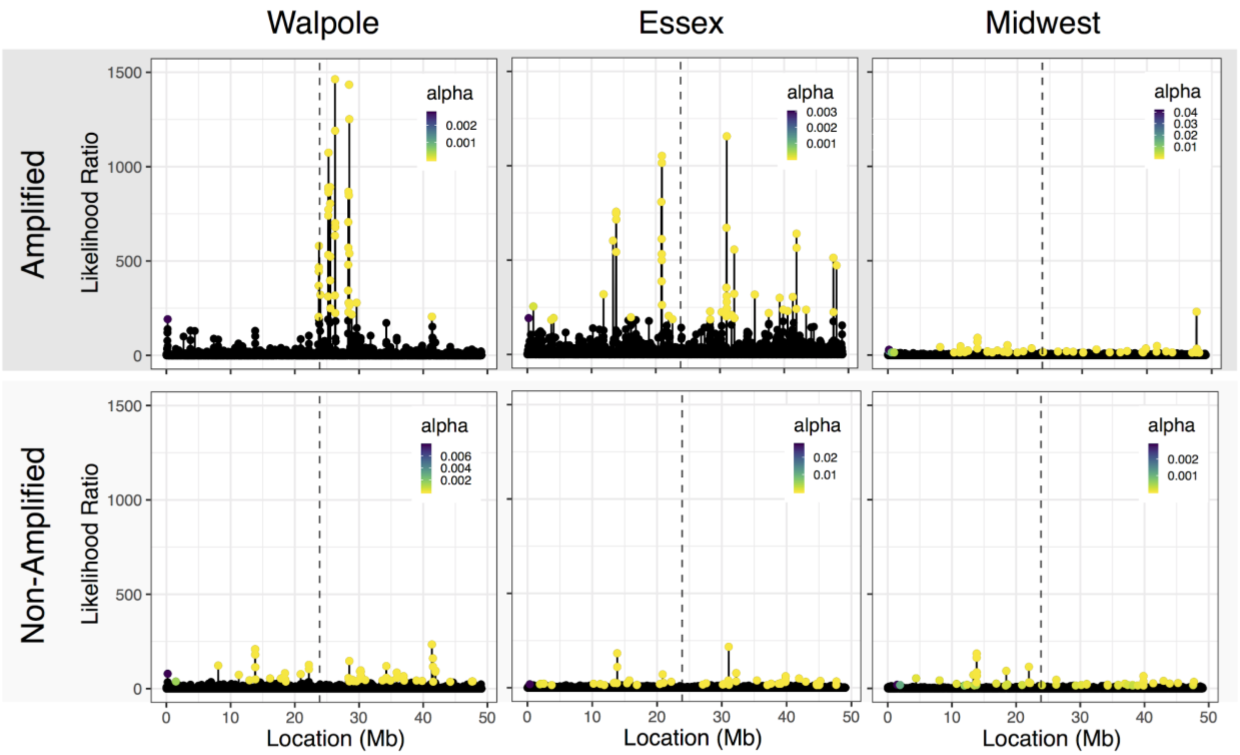

Fig. S4. *Sweepfinder2* likelihood ratio scores of a selective sweep occurring across chromosome 5. Scores were controlled for recombination rate variation and the genome-wide neutral site frequency spectrum. Alpha refers to the relative strength of recombination vs. selection, with smaller values indicating stronger selection.

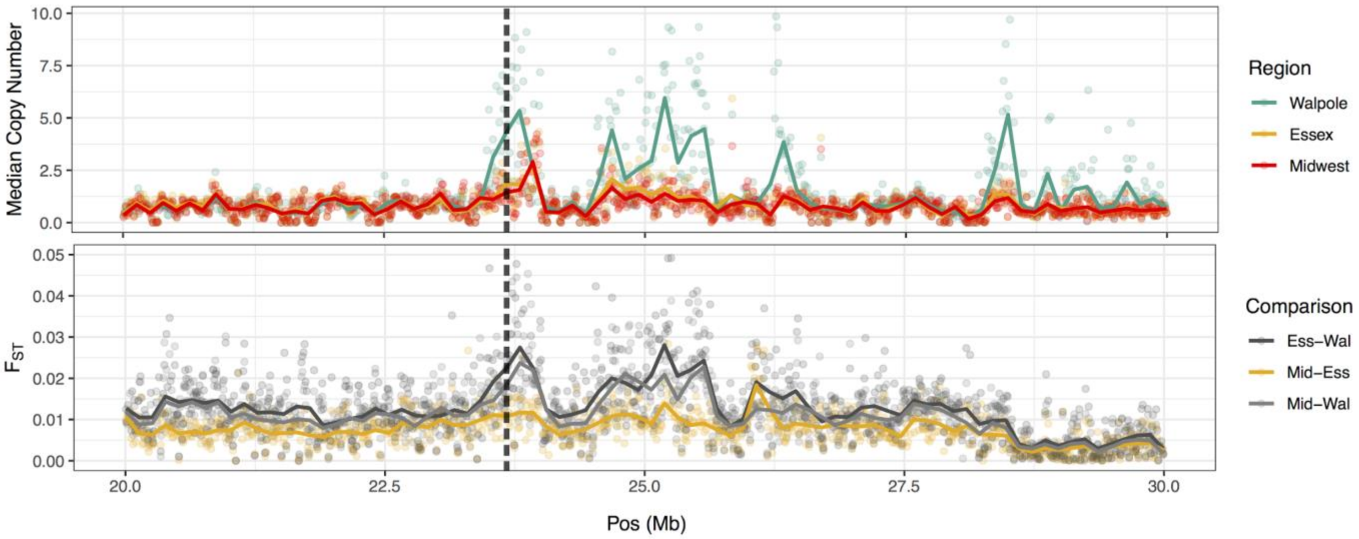

Fig. S5. Genetic differentiation (F_{ST}) between agricultural regions and median copy number within agricultural regions in 10kb windows along the 20 Mb - 30 Mb region of chromosome 5. EPSPS indicated by the vertical dashed grey black line.

Table S1. Metrics of the raw and haplotype-reduced reference genome assemblies.

Assembly	Total Size (Mb)	Sequence (#)	N50 (bp)	Longest sequence (bp)	Completeness (%)	Duplicates (%)
Raw Assembly	1,159,758,700	4,207	905,938	9,167,955	90	71
Haploid Assembly	663,660,067	2,514	1,738,871	13,655,724	87	6

Table S2. Correlation of PC1 and PC2 (Fig. 1E) with both longitude and latitude. From 4 separately run ANOVAs.

**Longitude**			
**PC1**	r_2 = 0.05279	p = 0.00190	$F_{1,160}$= 9.973
**PC2**	r_2 = 0.7685	p = <2.2e$_{-16}$	$F_{1,160}$= 535.3
**Latitude**			
**PC1**	r_2 = 0.08058	p = 0.0001482	$F_{1,160}$= 15.11
**PC2**	r_2 = 0.6025	p = <2.2e$_{-16}$	$F_{1,160}$= 245.1
